# EHMTI-0304. Headache determines quality of life in idiopathic intracranial hypertension

**DOI:** 10.1186/1129-2377-15-S1-C57

**Published:** 2014-09-18

**Authors:** A Sinclair, Y Mulla, K Markey, J Mitchell, S Patel

**Affiliations:** 1 Neurotrauma and Neurodegeneration School of Clinical and Experimental Medicine College of Medical and Dental Sciences, University of Birmingham, Birmingham, UK; 2 Birmingham Clinical Trials Unit, University of Birmingham, Birmingham, UK

## Introduction

No previous studies have assessed quality of life (QoL) in idiopathic intracranial hypertension (IIH) associated with a therapeutic weight loss. Our previously published prospective cohort study confirmed that weight loss significantly reduced intracranial pressure (ICP) and treated chronic active adult IIH.

## AIMS

Evaluate if QoL improved following treatment of IIH achieved through weight loss. Additionally, evaluate the relationship between QoL and changes in clinical outcome measures (body mass index (BMI), ICP (lumbar puncture), papilloedema (optical coherence tomography retinal nerve fibre layer thickness), automated perimetry (Humphrey visual field 24-2), LogMar visual acuity as well as headache severity (diarised verbal rating scale 0-10) and disability (headache impact test-6).

## Method

QoL was assessed using the short form 36 questionnaire (SF-36) before and after a dietary intervention and compared to changes in clinical outcomes. Baseline QoL was also compared to a control cohort.

## Results

At baseline, SF-36 scores were worse in IIH group compared to an age-matched population. Following therapeutic weight loss, with reduction of ICP (p<0.001), SF-36 significantly improved (8 out of 11 domains). Despite a significant improvement with therapeutic weight loss, the following were not associated with enhanced quality of life: ICP, papilloedema, automated perimetry, visual acuity and BMI. The only variables that significantly correlated with improved QoL were headache severity and disability, p<0.001.

## Conclusion

QoL significantly improved in IIH following therapeutic weight loss with reduction in ICP. Improvement in headache was the only factor that correlated with enhanced QoL. Effective headache management is a vital determinant of QoL in IIH.

No conflict of interest.

